# Dynamic FDG-PET imaging for differentiating metastatic from non-metastatic lymph nodes of lung cancer

**DOI:** 10.3389/fonc.2022.1005924

**Published:** 2022-11-10

**Authors:** Xieraili Wumener, Yarong Zhang, Zhenguo Wang, Maoqun Zhang, Zihan Zang, Bin Huang, Ming Liu, Shengyun Huang, Yong Huang, Peng Wang, Ying Liang, Tao Sun

**Affiliations:** ^1^ Department of Nuclear Medicine, National Cancer Center, National Clinical Research Center for Cancer, Cancer Hospital & Shenzhen Hospital, Chinese Academy of Medical Sciences and Peking Union Medical College, Shenzhen, China; ^2^ Paul C. Lauterbur Research Center for Biomedical Imaging, Shenzhen Institute of Advanced Technology, Chinese Academy of Sciences, Shenzhen, China; ^3^ Shenzhen Middle School, Shenzhen, China

**Keywords:** dynamic imaging, PET/CT, ^18^F-FDG, lung cancer, lymph Node

## Abstract

**Objectives:**

^18^F-fluorodeoxyglucose (FDG) PET/CT has been widely used in tumor diagnosis, staging, and response evaluation. To determine an optimal therapeutic strategy for lung cancer patients, accurate staging is essential. Semi-quantitative standardized uptake value (SUV) is known to be affected by multiple factors and may fail to differentiate between benign and malignant lesions. Lymph nodes (LNs) in the mediastinal and pulmonary hilar regions with high FDG uptake due to granulomatous lesions such as tuberculosis, which has a high prevalence in China, pose a diagnostic challenge. This study aims to evaluate the diagnostic value of the quantitative metabolic parameters derived from dynamic ^18^F-FDG PET/CT in differentiating metastatic and non-metastatic LNs in lung cancer.

**Methods:**

One hundred and eight patients with pulmonary nodules were enrolled to perform ^18^F-FDG PET/CT dynamic + static imaging with informed consent. One hundred and thirty-five LNs in 29 lung cancer patients were confirmed by pathology. Static image analysis parameters including LN-SUVmax, LN-SUVmax/primary tumor SUVmax (LN-SUVmax/PT-SUVmax), mediastinal blood pool SUVmax (MBP-SUVmax), LN-SUVmax/MBP-SUVmax, and LN-SUVmax/short diameter. Quantitative parameters including K_1_, k_2_, k_3_ and K_i_ and of each LN were obtained by applying the irreversible two-tissue compartment model using in-house Matlab software. K_i_/K_1_ was computed subsequently as a separate marker. We further divided the LNs into mediastinal LNs (*N*=82) and pulmonary hilar LNs (*N*=53). Wilcoxon rank-sum test or Independent-samples T-test and receiver-operating characteristic (ROC) analysis was performed on each parameter to compare the diagnostic efficacy in differentiating lymph node metastases from inflammatory uptake. *P<*0.05 were considered statistically significant.

**Results:**

Among the 135 FDG-avid LNs confirmed by pathology, 49 LNs were non-metastatic, and 86 LNs were metastatic. LN-SUVmax, MBP-SUVmax, LN-SUVmax/MBP-SUVmax, and LN-SUVmax/short diameter couldn’t well differentiate metastatic from non-metastatic LNs (*P*>0.05). However, LN-SUVmax/PT-SUVmax have good performance in the differential diagnosis of non-metastatic and metastatic LNs (*P*=0.039). Dynamic metabolic parameters in addition to k_3_, the parameters including K_1_, k_2_, K_i_, and K_i_/K_1_, on the other hand, have good performance in the differential diagnosis of metastatic and non-metastatic LNs (*P*=0.045, *P*=0.001, *P*=0.001, *P*=0.001, respectively). For ROC analysis, the metabolic parameters K_i_ (AUC of 0.672 [0.579-0.765], sensitivity 0.395, specificity 0.918) and K_i_/K_1_ (AUC of 0.673 [0.580-0.767], sensitivity 0.570, specificity 0.776) have good performance in the differential diagnosis of metastatic from non-metastatic LNs than SUVmax (AUC of 0.596 [0.498-0.696], sensitivity 0.826, specificity 0.388), included the mediastinal region and pulmonary hilar region.

**Conclusion:**

Compared with SUVmax, quantitative parameters such as K_1_, k_2_, K_i_ and K_i_/K_1_ showed promising results for differentiation of metastatic and non-metastatic LNs with high uptake. The K_i_ and K_i_/K_1_ had a high differential diagnostic value both in the mediastinal region and pulmonary hilar region.

## Introduction

Lung cancer is the leading cause of cancer-related deaths worldwide. In China, it ranks first with a 30% mortality rate (1, 2). Accurate staging of lymph nodes (LNs) is an important prognostic factor and is critical for treatment planning of lung cancer (3). Because the 5-year survival for stage IA lung cancer patients reaches 92%, while the IVA stage drops to 10% (4). For N-staging, the 5-year survival for stage N0 reaches 56%, while the N3 stage drops to 6% (5). Therefore, early diagnosis and accurate stage play an important role in improving survival rates.

^18^F-fluorodeoxyglucose (FDG) positron emission tomography/CT (PET/CT) has been widely used in tumor diagnosis, staging, and response evaluation. The National Comprehensive Cancer Network (NCCN) recommends ^18^F-FDG PET/CT for clinical staging (6). However, ^18^F-FDG PET/CT has limited sensitivity and specificity for detecting metastatic mediastinal LNs of non-small cell lung cancer (NSCLC) (7, 8). Preceding Meta-analysis (9) concluded that the sensitivity of ^18^F-FDG PET/CT for mediastinal staging in NSCLC patients was 0.81(0.70-0.89) and the specificity was 0.79(0.70-0.87), but the contribution of endemic infectious disease areas remains to be discussed. Because, several benign FDG-avid LNs exist in granulomatous disease, tuberculosis, interstitial lung disease, and other infectious conditions including pneumonia (10, 11). Stephen et al. (12) concluded that, compared to non-endemic regions with endemic infectious disease, ^18^F-FDG PET/CT has a 16% lower average specificity in endemic regions (77% [73%-80%] vs. 61% [49%-72%]). In addition to that, the standard uptake value(SUV)as a semi-quantitative metabolic parameter is affected by multiple factors, such as scan time, blood glucose level, etc … Therefore, SUV measurement of FDG activity is sometimes challenging to discriminate between benign and malignant in regions with a high prevalence of tuberculosis and granulomatous lesions, such as China. Thus, reliable imaging biomarkers for N-staging in lung cancer are crucial.

Radiopharmaceutical distribution is a dynamic process that varies widely in diseases and individuals (13). Dynamic PET/CT(dPET/CT)continuously acquires imaging data over a certain time period. Based on proper kinetic modeling, absolute quantitative metabolic parameters can be obtained, e.g., net influx rate K_i_, tumor blood flow K_1_, phosphorylation rate k_3_, etc. (14). Compared to static PET/CT(acquired about 60 min after FDG injection), dPET/CT extracts physiological and biochemical parameters and better reveals the pathophysiological mechanisms of diseases. These parameters were proven to be able to differentiate between benign and malignant (14). Previous studies have confirmed the advantages of dPET/CT in the diagnosis of lung cancer and inflammatory lung lesions (15–19), but reports regarding FDG-avid LNs in lung cancer were seldom seen.

In the present study, we compared ^18^F-FDG PET/CT dynamic imaging with static imaging and investigated the value of their metabolic parameters in the differential diagnosis between metastatic and non-metastatic FDG-avid LNs, especially in the mediastinal and hilar regions.

## Materials and methods

### Patients

**Figure 1:**
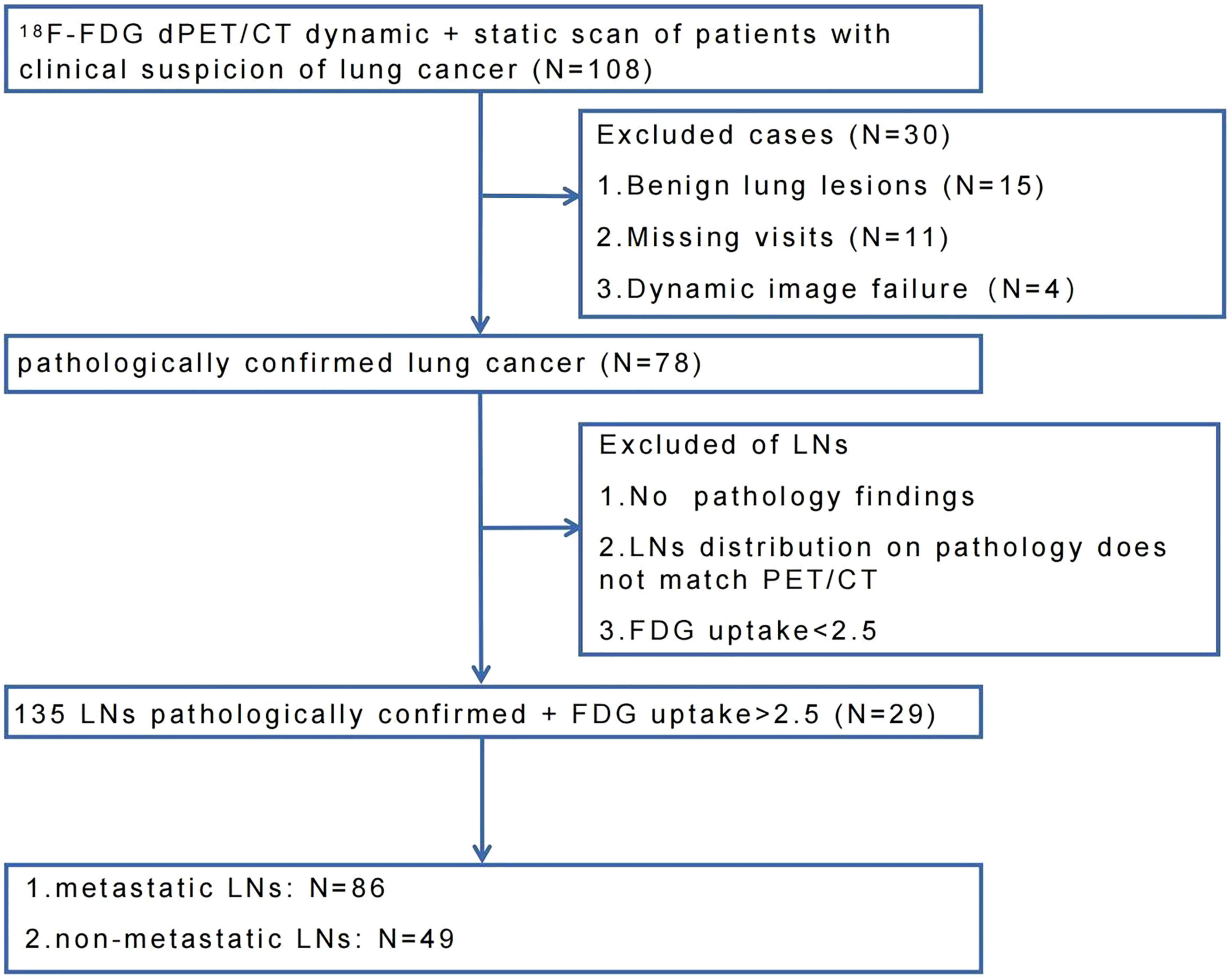
Enrollment flow chart of lung cancer patients and pathologically proven FDG-avid LNs.

This study was approved by the ethics committee of Cancer Hospital & Shenzhen Hospital, Chinese Academy of Medical Sciences (KYLH2022-1). A total of 108 patients were enrolled in this study from May 2021 to March 2022. All patients underwent chest CT and PET/CT scans for clinical suspicion of lung cancer without treatment. All patients signed a written informed consent before the PET/CT imaging. Four patients failed to finish dPET/CT scans due to physiological factors (e.g., failure to hold urine, nervous). Eleven patients were lost to follow-up. Ninety-three patients underwent puncture and/or surgery within two weeks after dPET/CT scan. Fifteen patients had benign pathological results, and seventy-eight patients were pathologically confirmed to have lung cancer. In these 78 lung cancer patients, LNs were excluded following the criteria: 1) SUVmax less than 2.5; 2) mismatch of distribution between PET/CT scan and puncture/surgery; 3) no proven pathology. Finally, 135 FDG-avid (SUVmax >2.5) LNs of 29 lung cancer patients were included and remarked, of which 86 LNs were confirmed metastatic and 49 LNs were confirmed non-metastatic. The flow chart of LNs enrollment was shown in **Figure 1**.

### PET/CT data acquisition and reconstruction

All patients fasted for at least 6 hours before the PET/CT scan (Discovery MI PET/CT, GE Healthcare, Milwaukee, USA). Blood glucose was maintained at lower than 8.0 mmol/L. The patients first underwent a whole-body CT scan from the head to the mid-femur in a supine position with the arms raised. The CT parameters were tube voltage of 120 kV, tube current setting of 10-220 mA, pitch of 1.375:1 and noise index of 20. The chest region PET scans were initiated immediately after the injection of ^18^F-FDG (264.8 ± 37 MBq)from an intravenous indwelling needle. The total dynamic scans lasted for 65 minutes. Dynamic scan data were partitioned into 28 frames as follows: 6 × 10 s, 4 × 30 s, 4 × 60 s, 4 × 120 s, 10 × 300 s. An additional whole-body static PET scan was performed at the end of the dynamic acquisition. The attenuation correction was performed using CT data, and reconstruction was performed using the Block sequential regularized expectation maximization reconstruction algorithm (BSREM) with 25 iterations and 2 subsets.

### PET/CT data analysis

Dynamic parameters of K_1_, k_2_, k_3_ and K_i_ were obtained based on the two-tissue irreversible compartment model. The image-derived input function (IDIF) was extracted from the ascending aorta by drawing a 10-mm-diameter ROI on six consecutive slices in an image obtained by combining early time frames (0–60 s), where the effects of motion and partial volume were less prominent than in the left ventricle. The uptake difference in blood and plasma was not accounted for in this study. In this model, we assumed unidirectional uptake of ^18^F-FDG (i.e., k_4_ = 0), with irreversible trapping in tissue as ^18^F-FDG-6-PO4 (20). Parametric images of each dynamic scan were generated using Voxel-based analysis. Given a large number of voxels in a whole-body image, the Lawson-Hanson non-negative least squares algorithm was applied to solve a linearized problem instead of the conventional nonlinear one (21). The 3D volume-of-interest (VOI) of each lesion was delineated using the semi-automatic methods with a threshold of 40% SUVmax in ITK-snap software (version 4.9). For the lesions with surrounding physiological uptake, 3D VOI was manually delineated slice-by-slice by two experienced nuclear medicine physicians. Then the segmented VOI was applied to the K_1_, k_2_, k_3_ and K_i_ parametric images to extract the quantitative measurements of each scan.

Static images were independently reviewed by two nuclear medicine physicians with more than 10 years of experience. LNs location was classified according to the International Association for the Study of Lung cancer (IASLC) lymph node map (22) and divided into the mediastinal region (zone 1 to 9) and pulmonary hilar region (zone 10 to 12). N-stage is based on the 8th edition of the TNM classification of lung cancer (23). LN-SUVmax and primary tumor (PT-SUVmax) were measured at the maximum cross-sectional level of the LNs and primary tumor. Mediastinal blood pool SUVmax (MBP-SUVmax) was measured by placing an ROI within the lumen of the aortic arch. The long diameter, short diameter, and CT density of the LNs were measured on a 5-mm CT image in the same axial direction as the SUVmax of LNs were measured.

Numerous previous studies have explored the threshold for LN positivity, including a SUVmax cut-off of 2.5 (24–28). The main purpose of this study was to further investigate the value of dPET/CT imaging for the differential diagnosis of high FDG uptake LNs. Therefore, in the context of previous studies of nodes and the stability and accuracy of the dynamic processing software, we considered SUVmax > 2.5 (axial images) as FDG-avid LNs. In case of disagreement between the two raters, the consensus was reached by discussion.

### Pathological evaluation

Two independent pathologists (over ten years of experience in lung cancer pathology) evaluated samples of dissected tissue. All dissected tumors and lymph nodes were sectioned and examined conventionally using hematoxylin-eosin staining. Immunohistochemical staining was also performed at the pathologist’s discretion.

### Statistical analysis

As the data distribution was non-normal, quantitative metrics of LNs were compared between groups using Wilcoxon rank-sum test or Independent-samples T-test. The receiver-operating characteristic (ROC) analysis was performed on each parameter to reveal the diagnostic efficacy in differentiating nonmetastatic and metastatic LNs with high FDG uptake. The difference in the area under curve (AUC) was determined by Delong’s test. A *P*-value less than 0.05 were considered statistically significant. All statistical analyses were performed in R statistical software (version 4.1.1).

## Results

### Characteristics of the patients and LNs

Patient and LNs characteristics were presented in **Table 1**. Among the 29 patients who underwent dPET/CT imaging, the average age was 62.0 (62.0 ± 10.20) years, and the number of male and female patients was 17 (58.60%) and 12 (41.40%), respectively. The pathological types of the primary tumor were adenocarcinoma in 17 patients (58.60%), squamous cell carcinoma in 9 patients (32.14%), small cell carcinoma in 2 patients (6.90%) and atypical carcinoid tumor in 1 patient (3.45%).

**Table 1:** Characteristics of the patients and LNs.

Characteristic	Distribution
**Sex** Male	17 (58.60%)
**Age (years)** Mean ± SD	62.0 ± 10.20
**Lobar distribution of the primary tumor(*N*)**	**29**
RUL/RML/RLL	4 (13.80%)/5 (17.20%)/3 (10.30%)
LUL/LLL	9 (31.0%)/8 (27.60%)
**Histopathological type of the primary tumor(*N*)**	**29**
Adenocarcinoma	17 (58.60%)
Squamous cell carcinoma	9 (32.14%)
Small cell carcinoma	2 (6.90%)
Atypical carcinoid tumor	1 (3.45%)
**Histopathological type of the LNs (*N*)**	**135**
**Non-metastatic group(*N*)**	**49**
Cancer-free	49 (37.12%)
**Metastatic group(*N*)**	**86**
Adenocarcinoma	60 (44.44%)
Squamous cell carcinoma	14 (10.61%)
Small cell carcinoma	12 (9.09%)
**LNs distribution(*N*)**	**135**
mediastinal LNs	82 (60.74%)
pulmonary hilar LNs	53 (39.26%)

SD standard deviation, RUL right upper lobe, RML right middle lobe, RLL right lower lobe, LUL left upper lobe, LLL left lower lobe.

Among the 135 LNs pathologically confirmed, 49 were non-metastatic (49/37.12%) and 86 were metastatic including 60 (44.44%) adenocarcinoma, 14 (10.61%) squamous cell carcinoma, and 12 (9.09%) small cell carcinoma. The LNs were divided into the mediastinal region (82/60.74%) and the pulmonary hilar region (53/39.26%) according to their distribution.

### PET/CT parameter analysis of FDG-avid LNs

**Figure 2:**
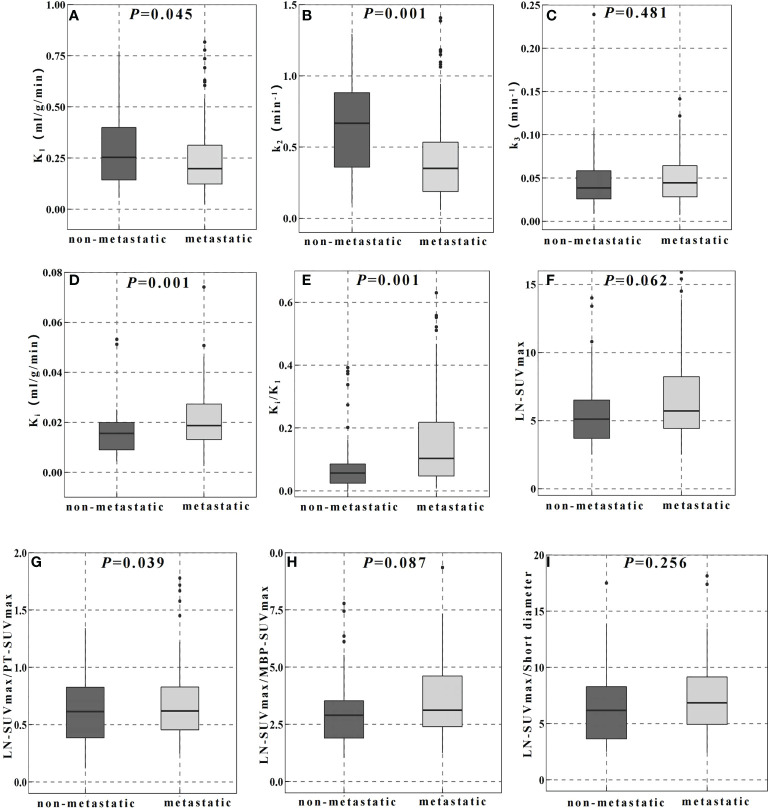
Comparison of dynamic **(A–E)** and static **(F–I)** PET/CT metabolic parameters in the differential diagnosis of FDG-avid LNs.

**Table 2** showed the parameter analysis of FDG-avid LNs in both dPET/CT and static PET/CT. In static PET/CT, LN-SUVmax/PT-SUVmax was statistically significant to differentiate non-metastatic and metastatic LNs (0.600 [ ± 0.304] vs 0.730 [ ± 0.411], *P*=0.039, **Figure 2G**). The CT density, length diameter, short diameter, as well as LN-SUVmax (**Figure 2F**), MBP-SUVmax, LN-SUVmax/short diameter (**Figure 2I**), and LN-SUVmax/MBP-SUVmax (**Figure 2H**) in static PET/CT, could not well differentiate metastatic and non-metastatic LNs (*P*>0.05) **Table 3**.

**Table 2:** FDG-avid LNs PET/CT characteristics and parameters analysis.

Characteristic	Non-metastatic group (*N*=49)	Metastatic group (*N*=86)	*P* Value
**Density (HU)**	43.00 [34.00;57.00]	38.00 [30.00;49.00]	0.147
**Length diameter (cm)**	1.10 [1.00;1.40]	1.10 [1.00;1.50]	0.292
**Short diameter (cm)**	0.80 [0.70;1.00]	1.00 [0.80;1.10]	0.057
**LN-SUVmax**	5.10 [3.70;6.50]	5.70 [4.43;8.23]	0.062
**MBP-SUVmax**	2.00 [1.70;2.00]	1.90 [1.70;2.20]	0.293
**LN-SUVmax/PT-SUVmax**	0.600 ( ± 0.304)**^▲^**	0.730 ( ± 0.411)**^▲^**	0.039^*^
**LN-SUVmax/MBP-SUVmax**	2.900 [1.900;3.529]	3.114 [2.402;4.608]	0.087
**LN-SUVmax/Short diameter**	6.167 [3.643;8.286]	6.854 [4.925;9.150]	0.256
**K_1_ (ml/g/min)**	0.253 [0.145;0.405]	0.205 [0.123;0.318]	0.045^*^
**k_2_ (min^-1^)**	0.666 [0.359;0.882]	0.350 [0.187;0.533]	0.001^*^
**k_3_ (min^-1^)**	0.039 [0.027;0.059]	0.044 [0.028;0.064]	0.481
**K_i_ (ml/g/min)**	0.016 [0.009;0.020]	0.019 [0.013;0.028]	0.001^*^
**K_i_/K_1_**	0.056 [0.024;0.085]	0.104 [0.048;0.250]	0.001^*^

^**▲**^LN-SUVmax/PT-SUVmax values was expressed as mean ± standard deviation (Independent-samples T-test), and the remaining indicators were expressed as median (interquartile spacing, Wilcoxon rank-sum test).

*stands for statistically significant difference.

**Table 3:** Diagnostic efficacy of PET/CT metabolic parameters.

Characteristic	Sensitivity	Specificity	cut−off value	AUC[95% CI]	*P* Value */*P* Value ▲
**SUV**max	0.826	0.388	4.050	0.596 [0.498-0.696]	–
**LN-SUVmax/PT-SUVmax**	0.999	0.204	0.236	0.566 (0.462-0.670)	–
**K_1_ (ml/g/min)**	0.581	0.612	0.234	0.604 [0.582-0.772]	0.910/0.602
**K_2_ (min-1)**	0.767	0.571	0.549	0.677 [0.584-0.774]	0.144/0.088
**K_i_ (ml/g/min)**	0.395	0.918	0.022	0.672 [0.579-0.765]	0.059/0.026
**K_i_/K_1_**	0.570	0.776	0.093	0.673 [0.580-0.767]	0.115/0.838

*Delong’s test comparison with SUVmax. ^**▲**^Delong’s test comparison with LN**-**SUVmax/PT-SUVmax.

In dPET/CT, K_i_ and K_i_/K_1_ in non-metastatic group (0.016 ml/g/min and 0.056) were lower than those in metastatic group (0.019 ml/g/min and 0.104), and the differences were statistically significant (*P*=0.001, *P*=0.001, respectively, **Figures 2D, E**). K_1_ and k_2_ in non-metastatic group (0.253 ml/g/min and 0.666 min^-1^) were higher than those in metastatic group (0.205 ml/g/min and 0.350 min^-1^) with statistical significance (*P*=0.045, *P*=0.001, respectively, **Figure 2A, B**). However, the k_3_ did not show significant difference between groups (0.039 and 0.044 min^-1^, *P*>0.05, **Figure 2C**).

### The ROC curves and cut−off values for PET/CT metabolic parameters

B**y** ROC curve analysis, the LN-SUVmax/PT-SUVmax cut-off value of 0.236, AUC of 0.566 (0.462-0.670), sensitivity of 0.999 and specificity of 0.204, respectively.

**Figure 3:**
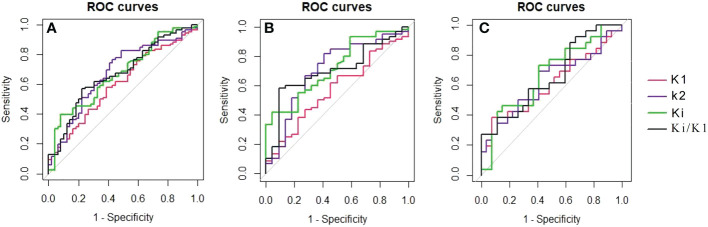
The ROC curves showed each metabolic parameter for the differential diagnosis of non-metastatic and metastatic FDG-avid LNs in all lesions **(A)**, the mediastinal region **(B)** and the pulmonary hilar region **(C)**.

In ROC curve analysis (**Figure 3A**), the cut-off value of K_1_, k_2_, K_i_ and K_i_/K_1_ were 0.234 (AUC of 0.604, sensitivity 0.581, specificity 0.612), 0.549 (AUC of 0.677, sensitivity 0.767, specificity 0.571), 0.022 (AUC of 0.672, sensitivity 0.395, specificity 0.918) and 0.093 (AUC of 0.673, sensitivity 0.570, specificity 0.776), respectively.

Delong’s test did not reveal any significant differences between SUVmax and other dynamic parameters including K_1_, k_2_, k_3_, K_i_, and K_i_/K_1_ (*P>*0.05). However, we performed Delong’s test with LN-SUVmax/P-SUVmax and found that the metabolic parameter Ki was statistically different (*P=*0.26).

### FDG-avid LNs analysis between the mediastinal region and pulmonary hilar region

According to the results of the second part of the statistics, LN-SUVmax/PT-SUVmax, K_1_, K_2_, K_i_, and K_i_/K_1_ were selected for further study. By ROC curve analysis in **Figure 2**, **3** in mediastinal region, the cut-off value of LN-SUVmax/PT-SUVmax, K_1_, k_2_, K_i_ and K_i_/K_1_ were 0.437 (AUC of 0.614 [0.453-0.775], sensitivity 0.833, specificity 0.500), 0.250 (AUC of 0.571, sensitivity 0.667, specificity 0.500), 0.526 (AUC of 0.705, sensitivity 0.817, specificity 0.591), 0.022 (AUC of 0.721, sensitivity 0.417, specificity 0.955) and 0.093 (AUC of 0.702, sensitivity 0.583, specificity 0.909), respectively. In pulmonary hilar region, the cut-off value of LN-SUVmax/PT-SUVmax, K_1_, k_2_, K_i_ and K_i_/K_1_ were 0.720 (AUC of 0.589 [0.431-0.747], sensitivity 0.577, specificity 0.677), 0.096 (AUC of 0.613, sensitivity 0.385, specificity 0.926), 0.662 (AUC of 0.623, sensitivity 0.692, specificity 0.593), 0.017 (AUC of 0.667, sensitivity 0.731, specificity 0.593) and 0.231 (AUC of 0.660, sensitivity 0.385, specificity 0.889), respectively.

The metabolic parameter K_i_ and K_i_/K_1_ have better diagnostic performance than other parameters both in the mediastinal region and pulmonary hilar region.

## Discussion

Clinical concerns have been raised for differential diagnosis of metastatic and non-metastatic LNs in N-staging of lung cancer to reduce the false-positive rate. In this study, we found the dPET/CT quantitative metabolic parameters K_1_, k_2_, K_i_ and K_i_/K_1_ had higher diagnostic efficacy than SUVmax.

The quantification of FDG uptake using SUVmax is the most used PET-derived parameter used as a biomarker of glucose metabolism for both diagnostic and follow-up purposes. However, metabolic parameters of dynamic PET/CT, such as K_i_ (the net influx rate), K_1_ (the surrogate of perfusion), k_2_ (the extraction) and k_3_ (the phosphorylated rate) recently emerged for diagnosis, differential diagnosis, and efficacy evaluation (29–32). Zhen et al. (33) concluded that, in a study of subcutaneous and *in situ* models of NSCLC in a mouse model, the dynamic metabolic parameter K_i_ could effectively differentiate between inflammation and tumor and could offer an assessment for inflammations at different locations of the body. Qing et al. (34) revealed that the dynamic metabolic parameter K_i_ had a higher AUC value than SUVmax in the differential diagnosis of benign and malignant pulmonary nodules, and that K_i_ could better distinguish benign from malignant nodules. These metabolic parameters more accurately represent the different stages of FDG metabolism and thus reflect the pathophysiological mechanisms of the disease. However, these previous studies have focused on primary tumors, and very few have been used to identify benign and malignant LNs.

Yang et al. (35) found in their lung cancer study, K_i_ value in the primary group of metastatic was higher than that of non-metastatic (0.050 ± 0.005 min^-1^ vs. 0.026 ± 0.004 min^-1^, *P*<0.001), and K_i_ value of metastatic LN in group metastatic was higher than that in group primary tumor (0.033 ± 0.005 min^-1^ vs. 0.016 ± 0.003 min^-1^, *P*<0.01). Kornelia Kajary et al. (36) found in their breast cancer study, the K_i_ values were higher in the positive LNs group than in the negative LNs group (0.039 vs. 0.023, *P*=0.0315), but K_1_, k_2_, k_3_ were not statistically different in the differential diagnosis of non-metastatic and metastatic LNs. In our study, compared to SUVmax, the dynamic metabolic parameters K_1_, k_2_, K_i_ and K_i_/K_1_ had higher differential diagnostic efficacy of metastatic and non-metastatic FDG-avid LNs in lung cancer. The K_i_ and K_i_/K_1_ values in the non-metastatic group were lower than those in the metastatic group. But the K_1_ and k_2_ values in the non-metastatic group were higher than those in the metastatic group. By ROC curve analysis, SUVmax had high sensitivity (0.826) but low specificity (0.388) in differentiating metastatic from non-metastatic FDG-avid LNs. On the contrary, K_i_ had high specificity (0.918), but low sensitivity (0.395). As a result, K_i_ seemed to be a good compensation for SUVmax. We considered the main reason for the low sensitivity of K_i_ is that we focused on LNs with FDG uptake > 2.5 and excluded the LNs with low uptake. In addition to that, among the 37 (37/49) non-metastatic FDG-avid LNs, the pathological type of the primary focus was adenocarcinoma. Whether dynamic metabolic parameters differ between pathological types requires further study.

To further improve the diagnostic efficacy of SUVmax, James Cerfolio first proposed the use of LN-SUVmax/PT-SUVmax to identify benign and malignant mediastinal LNs in lung cancer (37). Subsequently, Serra Fortuny et al. concluded that LN-SUVmax/PT-SUVmax is a good predictor of lymph node metastasis in NSCLC and the accuracy of mediastinal malignant LNs increased to 70% when using a 0.4 cut-off (38). Yang et al. (27) concluded that, when the LN-SUVmax/PT-SUVmax cut-off value was 0.200, the diagnosis of regional LN metastasis in NSCLC sensitivity of 83.30% and specificity of 71.30% (AUC of 0.780 [95% CI, 0.720-0.830]). In our study, LN-SUVmax/PT-SUVmax was statistically different in the differential diagnosis of metastatic and non-metastatic LN (*P*=0.039), with a trend similar to previous studies. However, for FDG-avid LNs, although there is a high sensitivity, the specificity is still low. In our study, when we tried to use the dynamic metabolic parameter K_i_/K_1_ ratio for prediction, we found that the K_i_/K_1_ also has good diagnostic efficacy, especially for in the hilar region LNs.

The density, short and long diameters derived from CT images have been studied frequently in the differential diagnosis of benign and malignant lesions. Dwamena et al. (39) conducted a meta-analysis, which included 29 studies (2226 patients) from 1990 to 1998, to evaluate the accuracy of CT in lung cancer mediastinal staging, with a sensitivity of 0.60 (95% CI, 0.58-0.62), specificity of 0.77 (95% CI, 0.75-0.79), and accuracy of 0.75 (95% CI, 0.74-0.76). However, the results of our study showed CT density, short diameter, and long diameter had no statistical difference (all *P>0.05*). Compared to CT imaging, the PET/CT improves the sensitivity and specificity of N-staging for lung cancer (40). However, both tumor and inflammatory lesions exhibit high FDG uptake (41, 42). Some lung cancer patients are often associated with underlying lung disease (e.g., tuberculosis, chronic obstructive pneumonia), especially in the elderly. Thus, in our study, the SUVmax had low specificity in FDG-avid LNs diagnosis, similar to the findings of previous studies (7, 8, 40).

Furthermore, previous studies have concluded that (24, 43, 44), SUVmax seemed to be more useful in predicting the benign and malignant LNs in the mediastinal area rather than the pulmonary hilar area. Lin et al. (43) also concluded that the total false positive rate could reach 70% when SUVmax predicted LNs status in the pulmonary hilar area, and most of the false positive cases were due to anthracosis, followed by reactive hyperplasia and hyalinized granuloma. In our study, by ROC curve analysis, K_i_ and K_i_/K_1_ had high differential diagnostic specificity both in the mediastinal region and pulmonary hilar region. Therefore, we believed that dynamic metabolic parameters, especially Ki, improved the accuracy of N-staging in the mediastinal and/or pulmonary hilar area, and provided a better complementary value than SUVmax.

**Figure 4:**
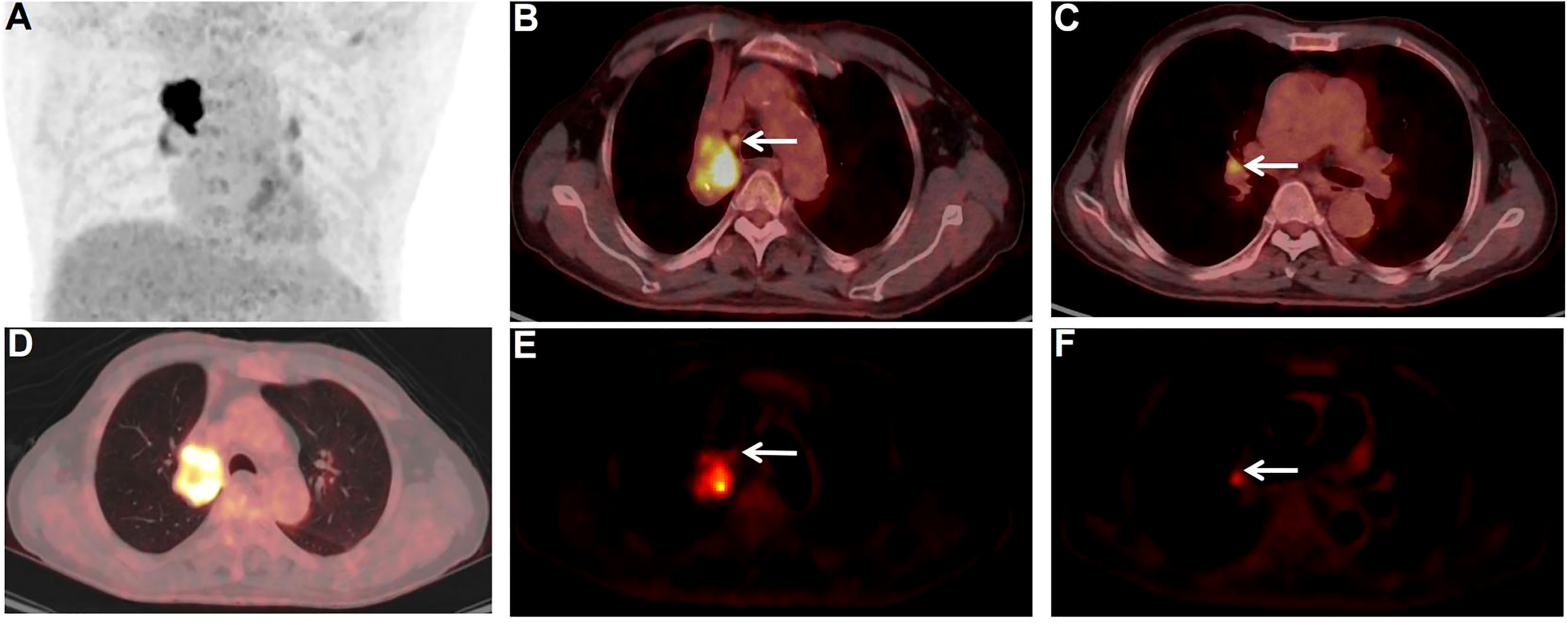
PET/CT images of non-metastatic and metastatic FDG-avid LNs. A 77-year-old male patient. Surgical pathology confirmed a squamous cell carcinoma in the upper lobe of the right lung. PET/CT scan showed multiple FDG-avid LNs in the mediastinal region and pulmonary hilar region. The FDG-avid LN (**E**, white arrow) in zone 4R (**B**, white arrow) of the mediastinal region was pathologically confifirmed to be cancer-free, with a size of 0.7×0.6 cm, SUVmax of 4.0, and Ki of 0.0084 ml/g/min. The other FDG-avid LN (**F**, white arrow) in zone 11R (**C**, white arrow) of the pulmonary hilar region was pathologically proven to be metastases, with a size of 0.7×0.7 cm, SUVmax of 4.9, and Ki of 0.0202 ml/g/min.

Interestingly in our study, N-stages in nine patients were re-staged when dynamic metabolic parameters were considered. Eight patients had a reduction in N-stage from N3/N2 to N1/N0 (shown in **Figure 4**), two had a reduction from N3 to N0 and two had a reduction from N3 to N1. One patient had an elevated N stage from the previous N0 to N1. Based on SUVmax alone, the FDG-avid LNs were hard to differentiate in this patient. However, dynamic metabolic parameters, especially K_i_, seemed promising for accurate N-staging of lung cancer.

Our study had several limitations. First, we excluded many patients without corresponding lesions in both PET/CT scans and puncture/surgery sites, thus this study was performed on relatively small sample size. Second, we collected fewer patients with benign lung lesions (tuberculosis, mechanized pneumonia), so we only studied FDG-avid LNs of lung cancer. Therefore, further study with expanded disease types and the sample size is needed to validate the current conclusion. Third, SUVmax rather than SUVmean was used in this study as we thought SUVmax was considered to be more stable and less affected by the partial volume effects (45–47). Fourth, motion correction was not considered in this study. It is known that motion in the chest region can affect not only the SUV but also the kinetic parameters quantification (48–51). Dedicated motion correction may be required to improve the accuracy of diagnosis in future studies.

## Conclusions

In addition to SUVmax, dynamic metabolic parameters demonstrated good complementary values in improving the accuracy of N-staging in lung cancer. The K_1_, k_2_, K_i_ and K_i_/K_1_ showed promising potential for differential diagnosis of FDG-avid LNs in lung cancer. The K_i_ and K_i_/K_1_ had a high differential diagnostic value both in the mediastinal region and pulmonary hilar region.

## Data availability statement

The original contributions presented in the study are included in the article/supplementary material. Further inquiries can be directed to the corresponding authors.

## Ethics statement

This study was approved by the ethics committee of Cancer Hospital & Shenzhen Hospital, Chinese Academy of Medical Sciences (KYLH2022-1). The patients/participants provided their written informed consent to participate in this study.

## Author contributions

XW and YL designed the project and write the manuscript. TS and ZW provided software, technical support and professional guidance. YZ analyzed data. ZZ, BH and SH organized data. YH and PW contribution FDG verification and injection. MZ and ML contribute to PET/CT scans. All authors contributed to the article and approved the submitted version.

## Funding

This research was funded by National Cancer Center, National Clinical Research Center for Cancer, Cancer Hospital & Shenzhen Hospital, Chinese Academy of Medical Sciences and Peking Union Medical College, Shenzhen (SZ2020MS008), Shenzhen High–level Hospital Construction Fund, and Shenzhen Science and Technology Innocation Committee (JCYJ20220531100209020).

## Conflict of interest

The authors declare that the research was conducted in the absence of any commercial or financial relationships that could be construed as a potential conflict of interest.

## Publisher’s note

All claims expressed in this article are solely those of the authors and do not necessarily represent those of their affiliated organizations, or those of the publisher, the editors and the reviewers. Any product that may be evaluated in this article, or claim that may be made by its manufacturer, is not guaranteed or endorsed by the publisher.
